# Infectivity of Aflagellar Epimastigotes of *Trypanosoma caninum* in the DH82 Cell Line and Mouse Peritoneal Macrophages

**DOI:** 10.1155/japr/7057514

**Published:** 2025-03-07

**Authors:** Kátia Cristina Silva Nascimento, Sandra Maria de Oliveira Souza, Aline Fagundes, Roger Magno Macedo Silva, Francisco Odencio Rodrigues de Oliveira Junior, Samanta Cristina das Chagas Xavier, Gilbert Q. Santos, Suzana Corte-Real, Juliana Helena da Silva Barros

**Affiliations:** ^1^Trypanosomatid Biology Laboratory, Oswaldo Cruz Institute, Oswaldo Cruz Foundation, Rio de Janeiro, Brazil; ^2^Structural Biology Laboratory, Oswaldo Cruz Institute, Oswaldo Cruz Foundation, Rio de Janeiro, Brazil; ^3^Clinical Research and Surveillance in Leishmaniasis Laboratory, Infectology National Institute, Oswaldo Cruz Foundation, Rio de Janeiro, Brazil; ^4^Rudolf Barth Electron Microscopy Platform, Oswaldo Cruz Institute, Oswaldo Cruz Foundation, Rio de Janeiro, Brazil; ^5^Celular Ultrastructure Laboratory de, Oswaldo Cruz Institute, Oswaldo Cruz Foundation, Rio de Janeiro, Brazil; ^6^Pedagogical Coordination Section, Army Complementary Training School and Salvador Military College, EsFCEx, Salvador, Brazil

**Keywords:** aflagellar epimastigote, amastigote, in vitro, infection, interaction, macrophagic cells

## Abstract

**Background:** *Trypanosoma caninum* presents aflagellar and flagellar epimastigote, trypomastigote, and spheromastigote forms in axenic cultures. Attempts to utilize trypomastigote forms of *T. caninum* to develop in vitro and in vivo infection models have failed. To investigate the infection potential of aflagellar epimastigotes, *T. caninum* interaction studies were performed using DH82 cells and BALB/c mouse peritoneal macrophages in Dulbecco's modified Eagle medium (DMEM)/F-12 medium supplemented with fetal bovine serum and bovine serum albumin. Light-field microscopy, scanning electron microscopy, and transmission electron microscopy were used to analyze these interactions. Regarding *T. caninum*–macrophage interactions, the following previously unseen results were obtained: (1) the aflagellar epimastigote form of *T. caninum* infects macrophages, and (2) *T. caninum* epimastigotes transformed into amastigotes inside macrophages. Aflagellar epimastigotes were seen adhering to and entering macrophages and differentiating to the amastigote form; amastigotes proliferated within the parasitophorous vacuole in macrophages after 15 min. At the final time point (48 h), there were few macrophages arranged on the coverslips, but interacting with free amastigotes of *T. caninum*, while some of the parasites changed to the flagellar epimastigote form. Considering the lack of information on *T. caninum* and its importance in public health, this study provides new insights into the biological cycle of *T. caninum* and parasite–host relationships.

## 1. Introduction

*Trypanosoma caninum* is a trypanosomatid flagellate that infects domestic dogs in leishmaniasis-endemic areas. To date, 62 cases of natural infection by *T. caninum* have been reported in dogs in six Brazilian states—Rio de Janeiro, São Paulo, Goiás, Minas Gerais, Mato Grosso, and Piauí—and the Federal District. *T. caninum* has been found in simple infections and in coinfection with *Leishmania infantum* and *Leishmania braziliensis* [1–5].

*T. caninum* appears to have low pathogenicity and has been isolated from the intact skin of healthy dogs [6, 7]. *T. caninum* establishment results from a reduced immunological response, as evidenced by the low serological titers (1/40, 1/80) of dogs positive for this parasite in intact skin cultures [6]. Notably, inflammatory infiltrates and amastigotes have not been observed in the tissues of a dog infected with *T. caninum* [8].

In axenic cultures of *T. caninum*, aflagellar and flagellar epimastigotes, trypomastigotes, and spheromastigotes have been observed [1, 7, 9]. Attempts have been made to use the flagellar epimastigote and trypomastigote forms of *T. caninum*, induced by metacyclogenesis, to infect cells in in vitro and in vivo experimental models. However, to date, attempts to experimentally infect Swiss Webster mouse macrophages, triatomine specimens of *Rhodnius neglectus* and *Triatoma infestans*, and epithelial cell lines with *T. caninum* have been unsuccessful [1, 9]. In this study, we hypothesized that the aflagellar epimastigote form of *T. caninum* was infective for DH82 macrophagic cells and BALB/c peritoneal macrophages because this atypical evolutionary form is present in axenic cultures of *T. caninum*, a condition that can stimulate the presence of evolutionary forms of the vector. Noteworthy, there are no reports of *T. caninum* being capable of infecting and differentiating into amastigote forms, as *Trypanosoma cruzi* does [1, 7, 10, 11]. The morphological diversity of the different species of trypanosomatids appears to be the result of the selective pressure of the environment, presented by the diversity in the length of the body and flagellum and width of the shape that can determine the pathogenicity of the parasite and its morphological change for its adaptation [12].

Our knowledge of the biological cycle of *T. caninum* is still very limited. To date, for example, the vector involved in the transmission of *T. caninum* is unknown, and whether this parasite infects other mammals, in addition to domestic dogs, is also unknown. Besides, the infectious evolutionary form of *T. caninum* in host cells remains unknown. Knowing the strategies to infect a host is an important step in understanding the nature of a parasite and how the parasitic interaction occurs [13]. In addition, the fact that *T. caninum* shares the same host with *Leishmania* spp. (domestic dogs) in areas endemic for leishmaniasis warrants further research on the biological cycle of *T. caninum*.

The macrophage-like cell line (DH82) is of hematopoietic origin from a 10-year-old male Golden Retriever diagnosed with malignant histiocytosis. The neoplastic progenitor cells were stabilized in 1985. This line presents macrophage morphology, with variation in size and quantity of the nucleus and nucleolus, in addition to being an excellent phagocytic cell. It is a heterogeneous cell line. It presents Fc surface receptors, important in cell–parasite interaction [14]. It has been used in different studies, including studies of bacteria [15, 16], fungi [17], viruses [18, 19], and *L. infantum* and *Leishmania amazonensis* [20]. Mendonça et al. [21] investigated the interaction between *T. cruzi* and the canine macrophage DH82 cell line for the first time, demonstrating its susceptibility to *T. cruzi* infection. The study observed the complete intracellular cycle, including parasite invasion, the presence of intracellular amastigotes, replication, transformation into trypomastigotes, and a prolonged infection period in dog macrophages. The authors reinforce the use of this cell as a model to investigate the host–parasite interaction in vitro and that it could contribute to experiments to identify new drugs for the treatment of Chagas disease.

This study investigated the interaction and infectious capacity of the aflagellar epimastigote form of *T. caninum* with DH82 and peritoneal macrophages. This work shows that the aflagellar epimastigote form of *T. caninum* can infect primary and lineage macrophages and reports the first observation of amastigotes as a developmental stage of *T. caninum*. Considering the lack of information about *T. caninum*, this study contributes to the understanding of its life cycle and of parasite–host relationships.

## 2. Materials and Methods

### 2.1. *T. caninum Isolate*

In this study, *T. caninum* isolate 12633 (COLTRYP 735)—deposited in the *Trypanosoma* Collection of Wild, Domestic and Vector Mammals (COLTRYP/FIOCRUZ) in 2017—was used. The sequence of this isolate has been deposited in GenBank under the Accession Number OP858808. This *T. caninum* isolate was obtained in 2013 from a fragment of intact dog skin in Rio de Janeiro, Brazil.

*T. caninum* was recovered from liquid nitrogen and transferred to a tube containing Novy–MacNeal–Nicolle (NNN) and Schneider's biphasic (NNN/Schneider) culture medium supplemented with 10% fetal bovine serum (FBS) and antibiotics (200 U penicillin + 100 *μ*g streptomycin) from Sigma–Aldrich at 27°C. After stabilization, the isolate was weekly inoculated in fresh medium.

### 2.2. Canine Macrophage–Like Cell Line DH82

The canine macrophage cell line DH82 (Code 0077) was obtained from the Cell Bank of Rio de Janeiro (https://bcrj.org.br). Cells were retrieved from liquid nitrogen and stabilized in a culture bottle in 3 mL of high-glucose Dulbecco's modified Eagle's medium (DMEM; LGC) supplemented with 10% FBS and antibiotics (200 U of penicillin + 100 *μ*g of streptomycin) (Sigma–Aldrich) at 37°C and 5% CO_2_. Adherent cells were dissociated using 1 mL of 0.25% trypsin (Gibco) for 10 min at 37°C. To inactivate trypsin, cold DMEM was added to the flask and centrifuged at 1899 × *g* for 10 min. The cell pellet was resuspended in DMEM at 37°C. Subsequently, this cell culture (0.5 mL) was mixed with fresh DMEM. Cells were maintained by passaging, according to cell confluence.

### 2.3. *T. caninum* and DH82 Cell Interactions

DH82 cells were washed with phosphate-buffered saline (PBS) and dissociated using trypsin. After dissociation, trypsin was inactivated with ice-cold (2°C–8°C) DMEM/nutrient mixture F-12 (DMEM/F-12) containing antibiotics (100 U penicillin + streptomycin; Gibco). DH82 cultures were centrifuged at 1899 × *g* for 10 min and resuspended in the same medium used for trypsin inactivation. DH82 cells were then counted in a Neubauer chamber; then, 4 × 10^4^ cells/mL were mixed with 300 *μ*L DMEM/F-12 with 100 U penicillin and streptomycin (Gibco) and seeded in 24-well culture plates, while 1 × 10^6^ cells/mL were mixed with 5 mL of the same medium and plated in 100-mm plates. After 1 h, the supernatant was removed from 100-mm plates and DMEM/F-12 supplemented with 10% FBS was added to half of these plates, whereas DMEM/F-12 supplemented with 2% bovine serum albumin (BSA) was added to the other half, and cells were incubated at 37°C and 5% CO_2_ for 24 h.

*T. caninum* parasites were then amplified in NNN/Schneider medium supplemented with 10% FBS and antibiotics (100 U penicillin and streptomycin; Gibco), transferred to 15 mL tubes, centrifuged at 1899 × *g* for 10 min, and resuspended in DMEM/F-12 containing antibiotics (100 U penicillin and streptomycin; Gibco). An aliquot was diluted 1:1000 in PBS and trypan blue to confirm the viability of the parasite. Amplified parasites were counted in a Neubauer chamber, and 10 parasites/DH82 cell (10:1) were inoculated in each well of the plate. Under all conditions and times, a control composed of cells without parasites was included.

### 2.4. *T. caninum* and Peritoneal Macrophage Cell Interaction

Peritoneal macrophages were obtained from the peritoneal lavage of female BALB/c mice weighing 18–20 g. Mice were euthanized using a combination of xylazine hydrochloride and ketamine hydrochloride. To collect peritoneal macrophages, approximately 10 mL of ice-cold DMEM/F-12 (Gibco) with 100 U penicillin and streptomycin (Gibco) was introduced into each animal's peritoneal cavity. The culture medium was aspirated and immediately placed on ice. The protocol for obtaining these cells was approved by the Ethics Committee on the Use of Animals (CEUA; License L-027/2018).

After collection, macrophages were counted in a Neubauer chamber and seeded at a concentration of 3 × 10^5^ cells/coverslip in 24-well plates in 300 *μ*L of DMEM/F-12 (Gibco) at 37°C and plated in 100-mm plates at a concentration of 2 × 10^6^ cells/plate to a total volume of 5 mL of the same culture medium. After 1 h, the culture medium was removed from 100-mm plates. In half of these plates, DMEM/F-12 with 10% FBS was added, whereas culture medium supplemented with 2% BSA was added to the other half. For the inoculum, *T. caninum* was treated as described above. After 24 h of incubation, peritoneal macrophages were inoculated with *T. caninum* at a density of 10 parasites per cell.

### 2.5. Experimental Design

Interaction experiments between *T. caninum* and either DH82 or peritoneal macrophages were performed in 24-well plates containing 13-mm glass coverslips for analysis by bright-field (BF) microscopy and scanning electron microscopy (SEM) and in 100-mm plates for analysis by transmission electron microscopy (TEM).

Interaction analyses by BF microscopy and SEM were performed in triplicate and duplicate, respectively, at 15, 30, and 45 min and 2, 6, 24, and 48 h. TEM analyses were performed at 45 min and 6 and 24 h, in duplicate. A control composed of cells without parasites was included for every condition and time. The methodologies used for BF microscopy, SEM, and TEM are described below.

### 2.6. BF Microscopy

Coverslips were washed with PBS and fixed with absolute methanol (Vetec) at each determined time point, stained with Giemsa (Merck) buffer for 2–4 min, and mounted with Entellan (Merck) on glass slides for analysis by BF microscopy under a Zeiss Axioplan microscope at a 100× magnification.

### 2.7. TEM and SEM

Coverslips and 100-mm plates were fixed with 2.5% glutaraldehyde diluted in 0.1 M cacodylate buffer (pH 7.2) + 3.5%sucrose + 2.5 mM CaCl_2_ for 1 h and washed three times with the same buffer. Lipid fixation was performed with 2% osmium tetroxide (OsO_4_) in 0.2 M cacodylate buffer (pH 7.2) + 7%sucrose + 5 mM CaCl_2_ for 30 min; then, the samples were washed three times with the same buffer. From this stage onward, the TEM and SEM protocols differed as follows.

#### 2.7.1. Steps for TEM

Sample dehydration was performed with increasing concentrations of acetone (30, 50, 70, 90, 100, 100, and 100%) for 10 min each, and the pellet was concentrated at 4500 rpm to remove the dehydrating agent. Subsequently, infiltration was performed overnight using a 1:1 solution of 100% acetone and resin (EPON Poly/Bed 812). The samples were then centrifuged to form a pellet, and the 1:1 acetone/resin solution was removed. Then, resin (EPON Poly/Bed 812) was added to the samples and polymerized in capsules for 72 h at 60°C.

The samples were processed into ultrathin sections, collected on copper gratings, and contrasted with uranyl acetate and lead citrate at the Electron Microscopy Platform of FIOCRUZ. Images were obtained using a TEM (JEOL JEM-1011) at the Rudolf Barth Electronic Microscopy Platform of the Instituto Oswaldo Cruz (IOC)/FIOCRUZ.

#### 2.7.2. Steps for SEM

After lipid fixation, the samples were dehydrated with increasing concentrations of ethanol (30, 50, 70, 90, 100, and 100%) for 10 min each. The samples were then subjected to critical point drying (a change in the physical state of CO_2_ inside the cell), metallization (coating of the sample with gold nanoparticles for electron release), and image acquisition using a SEM (JEOL JSM-6390LV) at the Rudolf Barth Electronic Microscopy Platform of the IOC/FIOCRUZ.

### 2.8. Statistical Analysis

Statistical analysis was performed using a random count of 200 macrophage cells on two coverslips, accounting for a total of 400 cells per repetition per experiment, by BF microscopy. Cell quantification was used to calculate the adhesion (macrophages with adhered parasites), infection (macrophages with internalized parasites), and adhesion and infection (macrophages in which the two events occurred) rates.

Quantification was also used to determine whether there was a difference in the number of parasites internalized by cells cultured in medium supplemented with FBS or those cultured in the presence of BSA and whether there was a difference in the number of parasites internalized between the two types of cells (DH82 and peritoneal macrophages).

In the present study, an experimental planning with two factors (sup_type and cell_type), each with two levels (sup_type: BSA and FBS, cell_type: DH82 and macrophage), was used. The level of significance adopted was 5%, and the following presupposition tests were performed: normality of residuals, homogeneity of variance, and autocorrelation. All statistical analyses were performed using R v.3.6.0 (2019 April 26).

## 3. Results

The interactions between *T. caninum* and peritoneal macrophages were similar to those between *T. caninum* and DH82 cells, with adhesion and infection events occurring simultaneously at all seven time points. At all these time points, adhesion of the aflagellar epimastigote form, infection, and interaction of the amastigote form with the cell occurred regardless of the supplementation conditions of the culture medium used. In addition, we observed that, when inoculated in the plates, the aflagellar epimastigote form of *T. caninum* immediately entered in contact with DH82 and peritoneal macrophages, despite the absence of flagella.

### 3.1. *T. caninum*–DH82 Interactions

**Figure 1:**
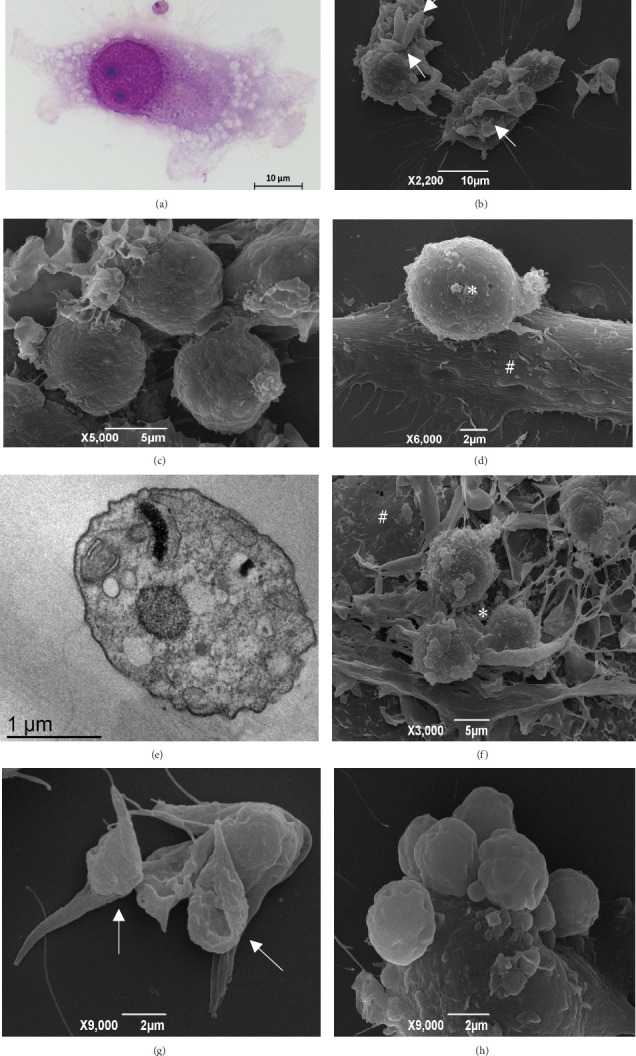
DH82 cell interactions with *T. caninum*. Fifteen minutes postinfection: (a) bright-field micrographies showing *T. caninum* amastigote (arrow); (b) SEM images showing *T. caninum* aflagellar epimastigotes (arrows) adhered to and interacting with DH82 macrophagic cells; 30 min postinfection: (c) SEM images showing amastigotes agglomerated; 2 h postinfection: (d) the interaction between an amastigote (∗) and a DH82 macrophagic cell (#); 6 h postinfection: (e) TEM image of a free amastigote; 24 h postinfection: (f) an apparent disruption of a macrophage (#) by amastigotes (∗); (g) changes suggestive of a transition from amastigote to epimastigote (arrow); 48 h postinfection: (h) SEM image of amastigote clusters interacting with DH82 macrophages.

Using BF microscopy, we observed that *T. caninum* adhered to the macrophages after 15 min of interaction. Free amastigotes (Figure 1(a)) as well as epimastigote and amastigote–DH82 macrophagic cell interactions were also observed, and the presence of vacuoles in the macrophages was noted. Using SEM, multiple adhesion patterns between aflagellar epimastigotes and DH82 cells were observed; these patterns included adhesion throughout the body and through the anterior or posterior portion of the parasite (Figure 1(b)).

**Figure 2:**
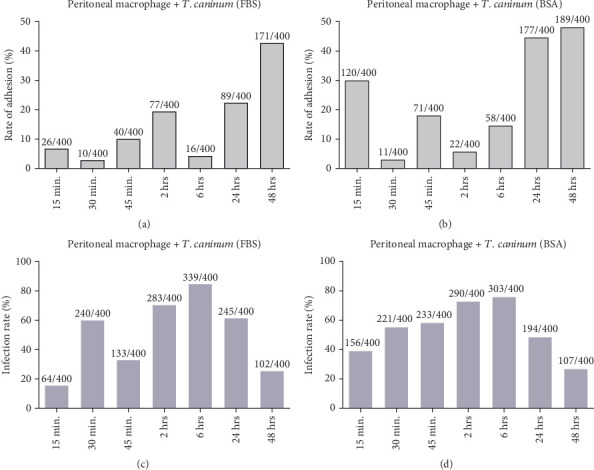
Adhesion and infection rates between murine macrophages and *T. caninum* under different culture conditions. Adhesion rates in medium supplemented with (a) FBS or (b) BSA. In the FBS condition, the highest adherence rates were reached at 2, 24, and 48 h postinoculation. In contrast, in the BSA condition, it was at 15 min and at 24 and 48 h postinoculation. (c, d) Infection rates of macrophages by *T. caninum* in culture medium supplemented with (c) SFB or (d) BSA. Under both conditions, infection rates reached their peaks at 6 h postinfection.

At 30 and 45 min postinoculation, several extracellular amastigotes were observed, forming clusters and in interaction with macrophagic cells. Using SEM, we observed the amastigote–macrophagic cell interaction (Figure 1(c)). Using TEM, further details of the amastigote form and of the interaction between the parasite and the macrophage could be seen. Two hours after the beginning of the experiment, free aflagellar epimastigotes in interaction with DH82 macrophages were observed, including interaction of the amastigote to macrophages (Figure 1(d)) and amastigote to aflagellar epimastigote shape changes. At 6 h, we observed, by SEM, the interaction between amastigotes and macrophages; apparently, amastigotes cause a disruption of DH82 cells. By TEM, we further observed amastigote structure details (flagellum completely retracted into the flagellar pocket, nucleus, kinetoplast, and oval shaped) (Figure 1(e)) and amastigotes within DH82 macrophagic cells. After 24 h, using SEM, we observed that amastigote clusters interacting with macrophage cells had apparently fractured these cells (Figure 1(f)). In addition, we detected shape changes suggesting a transition from the amastigote to the epimastigote forms (Figure 1(g)). By TEM, amastigotes within DH82 macrophagic cell were seen. At the last time point evaluated (48 h), we observed several free amastigotes and interactions between amastigotes and macrophages (Figure 2(a)). The formation of amastigote clusters was also observed (Figure 1(h)).

### 3.2. *T. caninum*–Peritoneal Macrophage Interactions

The interactions between *T. caninum* and peritoneal macrophages were similar to those between *T. caninum* and DH82 cells, with adhesion and infection events occurring at similar times and with similar kinetics. Notably, we observed peritoneal macrophage cell projections that interacted with the parasite. Moreover, immediately after inoculation, the aflagellar epimastigote form of *T. caninum* contacted macrophages, despite the absence of a flagellum.

**Figure 3:**
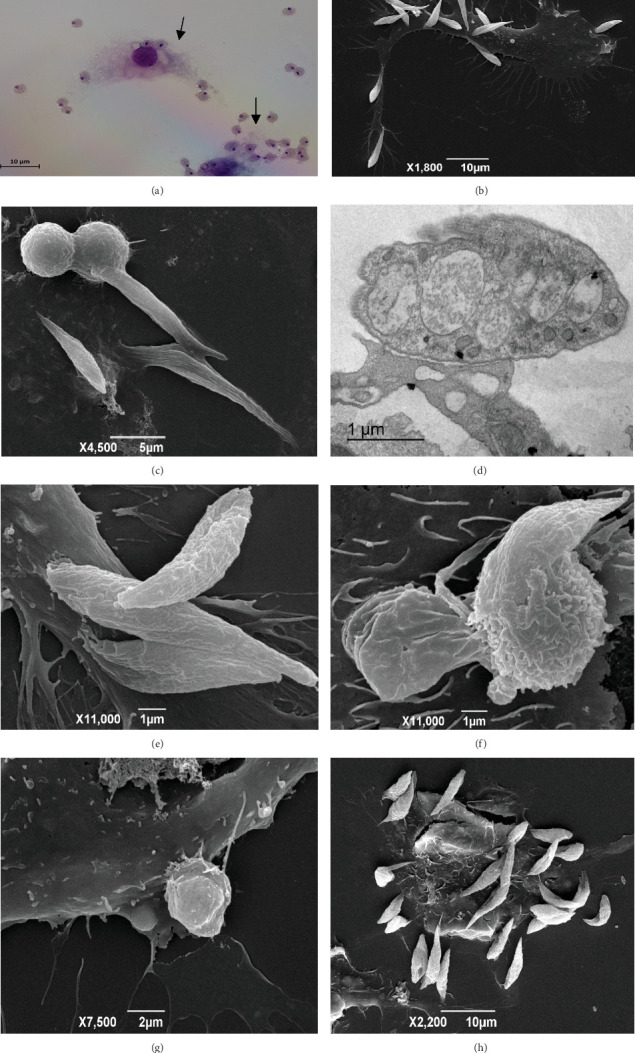
Murine peritoneal macrophage interactions with *T. caninum*. Fifteen minutes postinfection: (a) bright-field micrography of amastigotes (arrows) in the extracellular medium and interacting with BALB/c peritoneal macrophages. (b) SEM images showing aflagellar epimastigotes adhered to a BALB/c peritoneal macrophage; 30 min postinfection: (c) amastigotes and epimastigotes adhered to BALB/c peritoneal macrophages; 2 h postinfection: (d) TEM and (e) SEM images showing details of the adhesion of the parasite to BALB/c peritoneal macrophages; 6 h postinfection: (f) *T. caninum* changes from amastigote to aflagellar epimastigote; 24 h postinfection: (g) the interaction between amastigotes and BALB/c peritoneal macrophages; 48 h postinfection: (h) a cluster of aflagellar epimastigote forms adhered to a BALB/c peritoneal macrophage.

At 15 min, we observed free amastigotes (Figure 3(a)) and aflagellar epimastigotes interacting with macrophages and *T. caninum* changing from one evolutionary form to another in the extracellular environment. Using BF microscopy, after 30 min, we observed an increase in the number of amastigotes. Meanwhile, SEM revealed adhesion between macrophages and aflagellar epimastigotes (Figure 3(b)), interactions between aflagellar epimastigotes and amastigotes and peritoneal macrophages (Figure 3(c)), and the detailed structure of amastigotes. After 45 min, using BF microscopy, we observed interactions between amastigotes and macrophages; the presence of free amastigotes; and by, SEM, details of the adhesion of the parasite to the macrophages (Figure 3(e)). At 2 h, using TEM, we observed the adhesion of aflagellar epimastigotes to macrophages by examining cell projections in detail (Figure 3(d)). Elongated macrophages with numerous internalized amastigotes were also observed.

**Figure 4:**
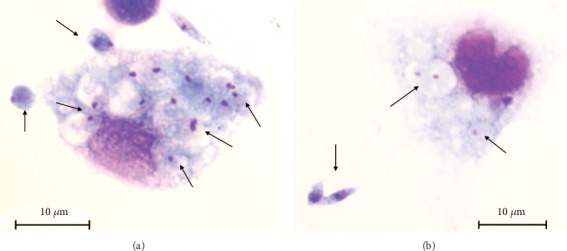
Murine peritoneal macrophage interactions with *T. caninum*. Six hours postinfection: bright-field microscopy technique Giemsa staining was performed at 100× magnification. Intracellular and macrophage membrane–adhered amastigotes (arrows) (a) and more than one amastigote in the same parasitophorous vacuole, indicating intracellular division (arrows) (b).

At 6 h, we observed free amastigotes and in interaction with macrophages, amastigotes that had been internalized in parasitophorous vacuoles (Figure 4(a)), and amastigotes within parasitophorous vacuoles inside macrophages (Figure 4(b)). SEM observations showed clusters of amastigotes—that were arranged above the cell or breaking it—and flagellar epimastigote forms next to the amastigotes. We also observed morphological changes in the parasite that led to the transformation of amastigotes into aflagellar epimastigotes (Figure 3(f)). At 24 h, we saw adhesion of aflagellar epimastigotes and interaction of amastigotes with macrophages as well as amastigotes inside the parasitophorous vacuole. Some details of the elongation of macrophage cell projections toward amastigotes were observed by SEM (Figure 3(g)). At 48 h, we observed an aflagellar epimastigote covered by cell projections and a cluster of aflagellar epimastigote forms adhered to the cell (Figure 3(h)). A predominance of aflagellar epimastigotes over amastigotes was observed.

### 3.3. Statistical Analysis

**Figure 5:**
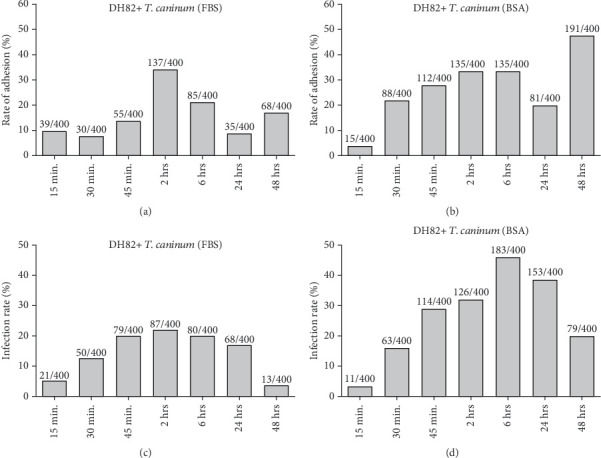
Adhesion and infection rates between DH82 cells and *T. caninum* under different culture conditions. Adhesion rates in medium supplemented with (a) FBS or (b) BSA. In the FBS condition, the peak of adherence was at 6 h postinoculation. In contrast, in the BSA condition, there was a progressive increase in adherence between 30 min and 6 h postinoculation, followed by a decrease and a subsequent increase, showing its highest peak of adherence at 48 h postinoculation. (c, d) Infection rates of DH82 cells by *T. caninum* in medium supplemented with (c) FBS or (d) BSA. In the condition with FBS, infection reached its highest rate at 2 h postinoculation; whereas, in the condition with BSA, the highest rate of infection occurred at 6 h postinoculation and then decreased.

An ANOVA statistical test was performed to compare the interaction of *T. caninum* with DH82 cells and peritoneal macrophages, after carrying out the normality, homogeneity, and autocorrelation tests, in which the proposals were accepted. The adherence and infection rates of *T. caninum* to the DH82 strain and peritoneal macrophages were calculated from a random count of 200 cells on two coverslips, for a total of 400 cells per experiment. The results are presented in the form of graphics in Figures 2 and 5. These results were used for statistical analyses.

There were no statistically significant differences in the number of parasites that adhered to the cells when comparing the interaction of *T. caninum* and DH82 cells and that with peritoneal macrophages (*p* = 0.498993). However, an analysis with two factors identified a significant difference among the adhesion results obtained from cultures supplemented with BSA or FBS (*p* = 0.006241) and in relation to the seven time points analyzed during each interaction experiment (*p* = 0.000607).

A significant difference was observed between the number of infected DH82 cells and that of mouse peritoneal macrophages (*p* = 1.93e − 14), and also between the number of infected cells in cultures with different medium supplements (*p* = 0.0157) in relation to the seven time points of each interaction experiment (*p* = 1.82e − 09).

We then investigated whether there were differences in the number of attached parasites and of infected cells if simultaneously analyzed using ANOVA. The results showed significant differences between the values of the two types of cells interacting with the parasite (*p* = 0.000333) as well as between those of the cultures supplemented with BSA and FBS (*p* = 0.001002) and those obtained in each of the seven time points of analysis for each interaction experiment (*p* = 0.003888).

Regarding the number of cells with internalized parasites, there was a difference when comparing the two cell types (*p* = 1.98e − 12) and between each of the seven time points analyzed for each interaction experiment (*p* = 1.88e − 06); however, there was no difference when comparing the means from the two types of medium supplementations (*p* = 0.309).

*T*-tests showed a higher mean interaction between *T. caninum* and peritoneal macrophages than between *T. caninum* and DH82 cells, regardless of whether the culture medium was supplemented with FBS or BSA. Notably, when comparing the two media, the interaction means were higher in the medium with BSA than in that with FBS in both DH82 and peritoneal macrophage cells. In general, higher means were observed in relation with *T. caninum*–peritoneal macrophage interactions in BSA-supplemented culture medium than with those in FBS-supplemented medium (Table 1).

## 4. Discussion

In this study, we tested the infective capacity of the aflagellar epimastigote form of *T. caninum* on macrophages. Since the description of *T. caninum* in 2009, studies have been conducted to understand how this parasite is present in nature and its ecological and epidemiological contexts. Since then, studies related to the life cycle and transmission of *T. caninum* have been performed using the trypomastigote form of the parasite after induction through metacyclogenesis, either to search for a vector response or cell infection in vitro [1, 9]. In previous experimental studies, interactions between the trypomastigote form of *T. caninum* and Swiss Webster mouse peritoneal macrophages or DH82 cells were not observed [1, 9]. In addition, in a study on natural infection of *T. caninum*, amastigote forms were not observed in histological sections of the skin of infected dogs [8]. Here, the following previously unknown information related to the biology of *T. caninum* was obtained: First, the aflagellar epimastigote form of *T. caninum* infects macrophages, and second, the amastigote form of *T. caninum* is observed in interaction with macrophages and can be described. We demonstrated the infective capacity of the aflagellar epimastigote form of *T. caninum*, which may be indicative of the mechanism of transmission of the infection between hosts. Aflagellar epimastigotes are an evolutionary form observed in all *T. caninum* isolates. The main features of the aflagellar epimastigote form are an empty flagellar pocket with no flagellum and a kinetoplast located lateral to the nucleus [7].

The *T. caninum* isolate used in this study, in axenic culture, showed an abundancy of aflagellar epimastigote forms of more than 90%. In addition, this isolate has a short generation time, and the concentration of aflagellar epimastigotes increases throughout the growth curve in axenic culture, reaching 100% after 20 days [22]. The same has been described for other *T. caninum* isolates, and trypomastigote and spheromastigote forms are rarely observed in axenic cultures [1, 7, 9]. The presence and maintenance of the aflagellar epimastigote form in the culture medium suggest that this form may play an important role in the life cycle of *T. caninum* in the host [23]. Epimastigote forms of *Trypanosoma* species show plasticity when changing from one evolutionary shape to another during their life cycle, exhibiting morphological diversity within the genus [24]. In axenic cultures in liver infusion tryptose (LIT), RPMI, and TC-100 media with 10% FBS, *Tribulus terrestris* presents an epimastigote form without free flagella, and studies have shown the infective capacity of epimastigotes of *T. cruzi* in vertebrates [25]. This corroborates with our data on the aflagellar epimastigote form of *T. caninum*.

When analyzing the interactions between *T. caninum* and both macrophagic cells, after 15 min, aflagellar epimastigotes were already adhered and free “rounded” parasites and interiorized amastigotes were observed. A few years after the description of *T. caninum* [1], we observed its amastigote evolutionary form for the first time. Surprisingly, the amastigote form was observed soon after the beginning of the interaction with macrophagic cells (15 min). Notably, other trypanosomatid species take longer to infect the cell, as described for the interaction between retinal cells and *L. amazonensis*, in which the parasite adheres to the cell in the first hour of interaction and is internalized after 6 h [26]. During the interaction between *T. cruzi* and DH82 cells, intracellular multiplication only occurs after 5 days [21].

After inoculation, we observed, under an inverted BF microscope, clusters of parasites, mainly aflagellar epimastigotes, that despite the absence of flagella moved toward adhered macrophages. It was also observed that *T. caninum* does not exhibit a pattern of adhesion to macrophages in the posterior or anterior part or throughout the body of the parasite. The same was observed by Uezato et al. [27], who reported that *Leishmania major* adheres to macrophages through different parts of its own body. In addition, Rittig et al. [28] demonstrated that the cell body parts of *Leishmania* spp. and *T. cruzi* trypomastigotes that bind to macrophages vary, depending on the species in the genus.

During *T. caninum*–murine macrophage interactions, we observed that the entry of the parasite into the cell occurred actively and/or through cellular projections around the parasite that either involved it completely or touched some part of its body. Although DH82 cells extend projections from one cell to another for maintaining communication, no cell projections toward the parasites were observed, suggesting that *T. caninum* adheres to DH82 cells in an attempt to actively enter these macrophages. These events have already been described for *T. cruzi*–macrophage interactions [29–31]. Macrophage projections on *L. major* were also observed by Uezato et al. [27].

Free amastigotes that interacted with cells were observed in this study. Our initial observations showed amastigotes in intimate contact with cells; later, by SEM and TEM, both evasion and cell invasion by these amastigote forms were seen. Similarly, a study by Ley et al. [32] demonstrated that the amastigote form of *T. cruzi* can sustain cell infection; in this study, trypomastigotes were differentiated into amastigotes and used to inoculate human monocytes in vitro, and 90% or more of these amastigotes of extracellular origin were absorbed by the cells. This finding was corroborated by Burleigh and Andrews [33] and, more recently, by Reignault et al. [34]. This information is consistent with our results, as we observed extracellular amastigotes interacting with macrophages, pointing to a new infection process. Most likely, *T. caninum* can maintain infection using this strategy, in the same manner as *T. cruzi*. However, further studies are required to determine, in detail, how this process occurs.

Knowing that the aflagellar epimastigote form of *T. caninum* is infective to mammalian cells under experimental conditions led us to consider the possibility that this is a strategy for the parasite to infect cells. This can occur because no energy expenditure is required to differentiate into the trypomastigote form. This fact suggests that *T. caninum* may be a parasite with high chances of infecting its host—in the same way as *T. cruzi*, in which epimastigote forms are infective. Epimastigote forms have long been considered noninfectious to host mammals, as corroborated by different studies [35, 36]. However, Kessler et al. [25] showed that the epimastigote forms of *T. cruzi* are resistant to the human complement system, interact with macrophages in a manner similar to that of trypomastigotes, and can also infect mice. Similarly, in vivo studies are required to confirm which infective form of *T. caninum* is involved in the biological cycle in vertebrate hosts.

Light and electron microscopy observations revealed the presence of two amastigotes of *T. caninum* within the same parasitophorous vacuole, suggesting division. Moreover, release of amastigotes from macrophages, change from amastigotes to aflagellar epimastigotes in the extracellular medium, and interactions between free amastigotes and cells were also observed. No change in the evolutionary form of the parasite was observed in the cell cytoplasm at any time, suggesting that evolutionary form changes are part of a parasite evasion strategy to deceive the host cell's immune system. Unlike *T. caninum* interaction characteristics, an interaction study between *Trypanosoma copemani* and PtK2 and Vero cells showed infection and interiorization of amastigotes, but no increase in the number of amastigotes or division was observed. The authors suggested that infection took place but that there might not be any intracellular cell cycle activation. Notably, *T. copemani* belongs to the same phylogenetic clade as *T. caninum* [37].

**Figure 6:**
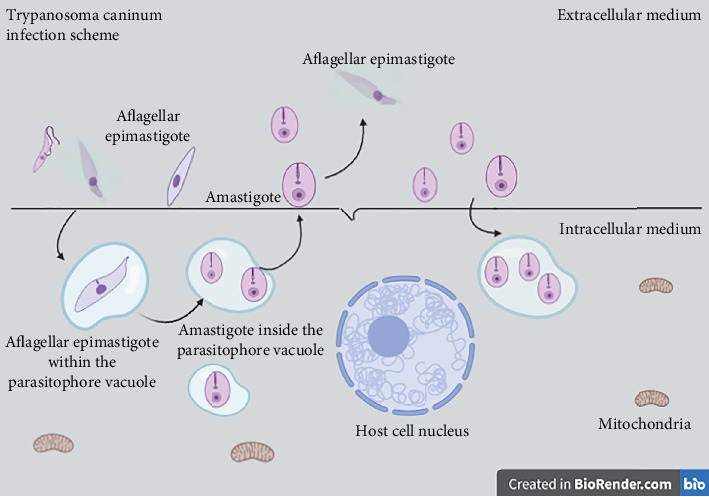
Schematic representation of the infection process. A parasite in the aflagellar epimastigote form adheres to a macrophagic cell either through macrophage projections or actively. Parasites then manage to enter the cell and remain in tight parasitophorous vacuoles in the intracellular environment. Within these vacuoles, aflagellar epimastigotes change to the amastigote form which multiplies inside the vacuoles. Soon after, the amastigotes break the cell and, in the extracellular environment, they differentiate into aflagellar epimastigotes or interact with the cell again in a new process of infection. The entire process takes 15 min. Over time, the vacuole becomes larger, and several amastigotes are visualized inside it (after 6 h). Within 48 h, the cells are quite broken, especially the peritoneal macrophages; however, in intact cells, there is great adhesion of aflagellar epimastigotes, and a new cycle of infection starts.

The experimental infection data presented here suggest that *T. caninum* infects mammalian host cells within 15 min by changing their evolutionary form, leading to changes in macrophage membrane receptors. These changes may explain the low immune response and the absence of detectable inflammatory infiltrates in histopathology [6, 8]. This is corroborated by studies suggesting that *T. caninum* is a parasite that does not harm canine hosts [1, 3]. When a parasite rapidly infects a cell and then changes its evolutionary form for a new attempt at infection, it can modulate different host responses, such as a change in different surface antigens; thus, different strategies can be used to invade host cells [31] (Figure 6).

Statistical analyses demonstrated successful cellular infection of *T. caninum* using media supplemented with BSA or FBS. *T*-tests showed that the higher means were obtained for those parasite–cell interactions (either with DH82 or murine macrophages) occurring in media supplemented which BSA. Therefore, the use of BSA can be an alternative to the use of FBS in culture media, not only because, during the experiments, all the corresponding (adhesion and infection) processes were observed, but because there were no significant differences in the number of parasites internalized among cells maintained in BSA- or FBS-supplemented media. Moreover, BSA has nutritional characteristics in its composition that do not promote large variations between batches, in opposition to those observed in the production of FBS, which is an important factor in the reproducibility of results [38, 39]. However, it is necessary to conduct experiments with other cell types and parasites and carry out new statistical tests to confirm whether the use of BSA can truly be a favorable substitute to FBS in experiments on cell–parasite infection.

## 5. Conclusions

In summary, we have shown that aflagellar epimastigotes are an infectious form of *T. caninum* in macrophagic cells and presented, for the first time, the amastigote form of *T. caninum.* These results provide new insights into *T. caninum* infection, which will help fill the gaps in our understanding of the biological cycle of this parasite. However, it is of importance to perform in vivo infection studies as well as to analyze the cell surface receptors that may be involved in the infection process to better understand the parasite–vertebrate host interaction process.

## Figures and Tables

**Table 1:** *T*-test in the comparison between cell types and types of culture medium supplementation: bovine serum albumin (BSA) and fetal bovine serum (FBS).

**Type of supplementation**	**Cell type**	**n**	**Mean**	**sd**
BSA	DH82	21	69.43	46.98
BSA	Peritoneal macrophage	21	142.29	70.07
FBS	DH82	21	39.62	23.15
FBS	Peritoneal macrophage	21	133.90	84.06

## Data Availability

The datasets supporting the results and conclusion of this article were included in the manuscript. The original dataset can be given upon request.
